# Reviewed article: Laroque MB, Tillmann TFF, Liskoski BV, Silva AER. Teledentistry actions in Primary Healthcare in Brazil during the COVID-19 pandemic – Cross-sectional study in 2021 Teledentistry and COVID-19. Epidemiol Serv Saude. 2025:34;e20240669.

**DOI:** 10.1590/S2237-96222025v34e20240669.a

**Published:** 2025-09-08

**Authors:** Maristela Honório Cayetano

**Table:** 

Rating	Excellent	Good	Average	Below average	Poor
Interest		✓			
Quality		✓			
Originality		✓			
Overall		✓			

**Table:** 

Questionnaire	Yes	No	Not applicable
Does the manuscript contain new and significant information to justify publication?	✓		
Does the Abstract (Summary) clearly and accurately describe the content of the article?	✓		
Is the problem significant and concisely stated?	✓		
Are the methods described comprehensively?	✓		
Are the interpretations and conclusions justified by the results?	✓		
Is adequate reference made to other work in the field?	✓		

## Manuscript Structure

Length of article is: Adequate

Number of tables is: Adequate

Number of figures is: Adequate

Please state any conflict(s) of interest that you have in relation to the review of this paper (state “none” if this is not applicable): None

## Comments

Não tem termo de consentimento livre e esclarecido (TCLE), explicar melhor a metodologia: se a pessoa que recebeu o link não concordar o restante do questionário não abre? /

Na linha 33, informa que na região Sul é oferecido o Telessaúde RS (esse serviço ainda encontra-se disponível? /

Na linha 20 há uma citação “utilização da teleodontologia” com a com o número 3 sobreescrito (acredito que não seja citação de autor das referências desse texto). Acredito que exista referências mais atuais, verificar / sugiro colocar as perguntas do questionário em anexo.

